# Supplementary material for: Facility ownership and mortality among older adults residing in care homes
Source: PLoS One. 2019 Mar 1;14(3):e0197789. doi: 10.1371/journal.pone.0197789 (PMC6396963; doi:10.1371/journal.pone.0197789)
Supplement: S1 File — (PDF) [file pone.0197789.s001.pdf]

APPENDIX

1. Supplementary methods

1.1. Types of facilities. Definitions as were used in this work

1.2. Complete description of the methods of mortality analysis

2. Supplementary results and tables

2.1. Sensitivity analysis further stratifying length of stay

2.2. S1 Table A. Selected Characteristics of Studies Showing Results of Ownership and Mortality in Nursing Homes

2.3. S1 Table B. Distribution of baseline characteristics by type of facility after standardization to the overall institutionalized population

* * *

1. Supplementary methods

1.1. Types of facilities. Definitions as were used in this work

These definitions apply to the reality of 1999. In the present moment, the situation is basically the same, but other types of organizations have come into the market, for example facilities whose property is public but managed by a private company.

Public. Facilities completely owned by a public administration (Government)

Private. Non-public (Facilities completely owned by a private organization).

Subsidized. Private facilities with a majority of their residents' fees/costs partly or wholly covered by a public administration. These facilities usually receive residents similar to that of public facilities. Thus they combine the type of resident that would be admitted into a public facility but with the characteristics of private management, although the contract agreements between the public administration and the private companies usually include clauses of quality that could be assimilated to the public residences.

A *non-profit* organization is an entity whose purpose is not the pursuit of an economic benefit but mainly pursues a social, altruistic or humanitarian purpose. These types of institutions are usually financed through grants and donations from individuals, companies, and institutions and organizations of all kinds. We included companies expressly defined as non-profit and those owned by religious organizations.

We characterized as *for-profit* those private companies for which we found no indication of non-profit status, and thus we assumed that they are organizations that include making money as one of their primary goals.

Some features of the Spanish long-term care system can be found in: *Damián J, Pastor-Barriuso R, Valderrama-Gama E. Descriptive epidemiology of undetected depression in institutionalized older people. J Am Med Dir Assoc. 2010;11(5):312-9* (Reference 23).

1.2. Complete description of the methods of mortality analysis

For mortality risk analyses, we used Kaplan-Meier methods and spline-based survival models[19] weighted by the above combined weights and stratified by type of facility. This allowed us to obtain nonparametric and smooth estimates of the mortality curves that would have been observed in the institutionalized population had every resident been cared for in each given type of facility[18]. Spline-based survival models parameterized stratum-specific log cumulative hazards as distinct natural cubic splines of log time with a single internal knot at the 50th percentile of the uncensored log time distribution[19]. Adding further internal knots did not improve model fit. We used these weighted spline-based survival models to estimate standardized differences in cumulative mortality at 2, 5, and 10 years of follow-up for each type of facility compared with large-sized public facilities (as reference) – with 95% confidence intervals (CIs) derived from robust standard errors of spline coefficients by applying delta methods. In addition to risk differences, we estimated standardized differences in median follow-up times (50% cumulative mortality) and their 95% CIs, comparing each type of facility with large-sized public facilities.

We evaluated homogeneity in risk differences across pre-specified subgroups of residents defined by baseline age (65–84 or ≥85 years), sex (woman or man), educational level (less than

primary, or primary, or more), length of stay (<3 or ≥3 years), dementia (yes or no), number of chronic conditions other than dementia (0–2 or ≥3), and functional dependency (no/mild or moderate/severe/total). This was done by fitting spline-based survival models weighted by combined weights and stratified by type of facility and resident subgroup. Combined weights for subgroup analyses were calculated as the product of sampling weights and subgroup-specific standardization weights, in order to standardize cumulative mortality curves in each type of facility and resident subgroup to the weighted distribution of confounders in the entire resident subgroup.[16] Standardized differences – in 5-year cumulative mortality and 95% CIs for each type of facility compared with large-sized public facilities – were estimated within each resident subgroup, and tested for homogeneity across subgroups by using joint Wald tests.

2. Supplementary results and tables

2.1. Sensitivity analysis further stratifying length of stay

In fully-adjusted models by inverse probability weighting with length of stay further stratified as <2, 2–4, and ≥5 years, the standardized 5-year mortality risk differences (95% CIs) for residents in medium-sized public/subsidized, medium-sized private, and small-sized private facilities compared with those in large-sized public institutions were −7.4% (−20.2% to 5.4%), −21.1% (−35.2% to −7.1%), and −13.4% (−26.2% to −0.6%) and the standardized differences (95% CIs) in the median survival time were 1.1 (−0.7 to 2.8), 3.9 (1.0 to 6.8), and 1.9 (0.1 to 3.7) years, respectively.

S1 Table A. Summary of the main characteristics of studies showing results of ownership and mortality in nursing homes.

Authors, publication year	**Survey year**	**Design**	**Country**	**Ownership**	**Follow up, years**	**Higher mortality**
Spector & Takada, 1991 [4]	1984-1986	Prospective cohort	Rhode Island, USA	FP, NP	0.5	NP
Bell & Krivich, 1990 [5]	1986-1987	Ecologic	Illinois, USA	Public, Private, FP, NP	1	Public [a]
Zinn et al, 1993 [6]	1987	Ecologic	Pennsylvania, USA	Private FP, NP	1	NP
Cohen & Spector, 1996 [7]	1987	Retrospective cohort	USA, National	Public, Private, FP, NP	1	Null
Spector, Selden & Cohen, 1998 [8]	1987	Retrospective cohort	USA, National	Private FP, NP	1	FP (only in non-Medicaid residents)
Bliesmer et al, 1998 [9]	1989; 1990; 1991	Retrospective cohort	Minnesota, USA	FP, NP	Three 1-year periods	NP
Porell et al, 1998 [10]	1991-1993	Retrospective cohort	Massachusetts, USA	FP, NP	2.67	Null
Intrator et al, 1999 [11]	1993	Prospective cohort	Ten States, USA	FP, NP	0.5	Null
McGregor et al, 2006 [12]	1996-1999	Retrospective cohort	British Columbia, Canada	Public, Private, FP, NP	3.3	Null
Tanuseputro et al 2015 [13]	2010-2012	Retrospective cohort New admissions	Ontario, Canada	Private, public FP, NP	1	FP

Note: FP, for-profit; NP, not-for-profit.
[a] Not possible to be adequately assessed because the confidence intervals were not reported.

S1 Table B. Distribution of baseline characteristics by type of facility after standardization to the overall institutionalized population in Madrid, Spain, 1998–1999.

Baseline characteristic	Overall [a]	Facility[b]				*P* value[c]
		Large-sized public	Medium-sized public/subsidized	Medium-sized private	Small-sized private	
Age (years)						0.98
65–74	13.2	13.4	11.6	9.2	13.0	
75–79	15.5	18.1	13.2	12.1	18.5	
80–84	24.1	24.8	25.9	24.4	25.3	
85–89	27.0	24.4	29.1	31.2	25.6	
≥90	20.2	19.3	20.2	23.1	17.6	
Sex						0.78
Women	74.5	73.5	68.6	74.6	74.3	
Men	25.5	26.5	31.4	25.4	25.7	
Educational level						0.70
Less than primary	42.3	40.8	45.4	47.1	38.0	
Primary or more	57.7	59.2	54.6	52.9	62.0	
Length of stay (years)						0.74
<3	47.0	49.1	46.5	49.0	55.7	
≥3	53.0	50.9	53.5	51.0	44.3	
Dementia						0.79
No	75.1	75.8	79.7	74.7	72.5	
Yes	24.9	24.2	20.3	25.3	27.5	
No. of chronic conditions						0.28
0–2	49.2	48.4	44.4	39.6	55.7	
≥3	50.8	51.6	55.6	60.4	44.3	
Functional dependency						0.91
No/mild	51.4	52.9	56.7	53.6	46.0	
Moderate	24.2	23.8	23.6	23.4	25.2	
Severe/total	24.4	23.3	19.7	23.0	28.8	

[a] Sampling-weighted percentages.
[b] Fully-weighted percentages taking into account both sampling and standardization weights.
[c] *P* value for homogeneity of fully-weighted percentages across types of facility.
